# *Staphylococcus aureus* bacteremia with iliac artery endarteritis in a patient receiving ustekinumab

**DOI:** 10.1186/s12879-016-1912-5

**Published:** 2016-10-20

**Authors:** Insa Joost, Johannes Steinfurt, Philipp T. Meyer, Winfried V. Kern, Siegbert Rieg

**Affiliations:** 1 Division of Infectious Diseases, Department of Medicine II, University Medical Center Freiburg, Hugstetter Str. 55, Freiburg, 79106 Germany; 2 Department of Cardiology and Angiology I, Heart Center, University of Freiburg, Hugstetter Strasse 55, Freiburg, 79106 Germany; 3 Department of Nuclear Medicine, University Medical Center Freiburg, Hugstetter Str. 55, Freiburg, 79106 Germany

**Keywords:** Immunosuppression, Biologicals, IL-12, IL-23, Intravascular infection

## Abstract

**Background:**

Ustekinumab (Stelara®), a human monoclonal antibody targeting the p40-subunit of interleukin (IL)-12 and IL-23, is indicated for moderate to severe plaque psoriasis and psoriatic arthritis. In large multicenter, prospective trials assessing efficacy and safety of ustekinumab increased rates of severe infections have not been observed so far.

**Case presentation:**

Here, we report the case of a 64-year old woman presenting with chills, pain and swelling of her right foot with dark maculae at the sole, and elevated inflammatory markers. She had received a third dose of ustekinumab due to psoriatic arthritis three days before admission. Blood cultures revealed growth of *Staphylococcus aureus* and imaging showed a thickening of the aortic wall ventral the bifurcation above the right internal iliac artery, resembling an acute bacterial endarteritis. Without the evidence of aneurysms and in absence of foreign bodies, the decision for conservative management was made. The patient received four weeks of antibiotic therapy with intravenous flucloxacillin, followed by an oral regime with levofloxacin and rifampicin for an additional four weeks. Inflammatory markers resolved promptly and the patient was discharged in good health.

**Conclusion:**

To our knowledge, this is the first report of a severe *S. aureus* infection in a patient receiving ustekinumab. Albeit ustekinumab is generally regarded as a safe drug, severe bacterial infections should always be included in the differential diagnosis of elevated inflammatory markers in patients receiving biologicals as these might present with nonspecific symptoms and fever might be absent. Any effort to detect deep-seated or metastatic infections should be made to prevent complications and to secure appropriate treatment. Although other risk factors for an invasive staphylococcal infection like psoriasis, recent corticosteroid injection, or prior hospitalisations were present, and therefore a directive causative link between the *S. aureus* bacteraemia and ustekinumab can not be drawn, we considered the reporting of this case worthwhile to alert clinicians as we believe that ongoing pharmacovigilance to detect increased risks for rare but severe infections beyond phase II and phase III trials in patients treated with biologicals is essential.

## Background

*Staphylococcus aureus* is one of the most common causative organisms of community-acquired or healthcare-associated bloodstream infections (BSI) [1, 2]. Inhouse mortality of *S. aureus* bacteremia (SAB) is considerably high (20–30 %) [3]. According to a population based study, patients with severe psoriasis have a higher risk for serious infections with significantly more hospitalizations for skin infections [4] compared to the reference population. Moreover, patients with psoriasis are more frequently colonized with *S. aureus* than the normal population, yet are not regarded as particularly susceptible to *S. aureus* infections, which may be due to a pronounced induction of innate defense antimicrobial peptides in psoriatic skin lesions [5, 6]. However, a small study reported the occurrence of *S. aureus* bacteremia in patients with extensive disease i.e. erythrodermic psoriasis [7].

A large prospective study in France in patients receiving TNF-α antagonists mainly due to rheumatoid or psoriatic arthritis, found *S. aureus* as the dominant causative pathogen of BSI, including all recorded deaths and intensive care admissions. The majority of patients with *S. aureus* BSI experienced subsequent osteoarticular manifestations (63 vs. 4 % in BSI patients due to other pathogens) [8].

Ustekinumab is a human monoclonal antibody that binds to the p40-subunits of interleukin (IL)-12 and IL-23 thereby preventing binding of these cytokines to their respective receptors and inhibiting T cell activation. Ustekinumab was approved by the FDA and EMA in 2009 for the treatment of adult patients with moderate to severe plaque psoriasis as well as patients with active psoriatic arthritis alone or in combination with methotrexate.

Like other immunosuppressive agents, ustekinumab may potentially increase the risk for acquiring infections or predispose to unusual or particularly severe manifestations of infections or to activate latent infections. Product labels of ustekinumab include warnings of serious infections and advise not to start the therapy during clinically important infections, to stop its application in case a serious infection develops and to use it with caution in patients with chronic or recurrent infections. A pre-treatment tuberculosis screen is recommended. However, in large multicenter trials infection rates were not elevated compared to other biologicals or non-biological immunosuppressive agents [9–14].

In the following article we report the case of a 64-year old woman receiving ustekinumab due to psoriatic arthritis who developed *S. aureus* bacteremia with iliac artery endarteritis.

## Case presentation

**Fig. 1:**
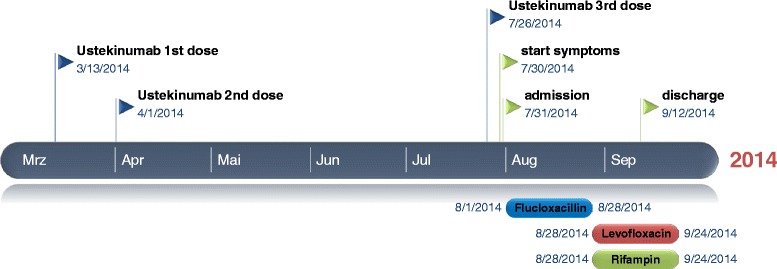
Timeline

**Fig. 2:**
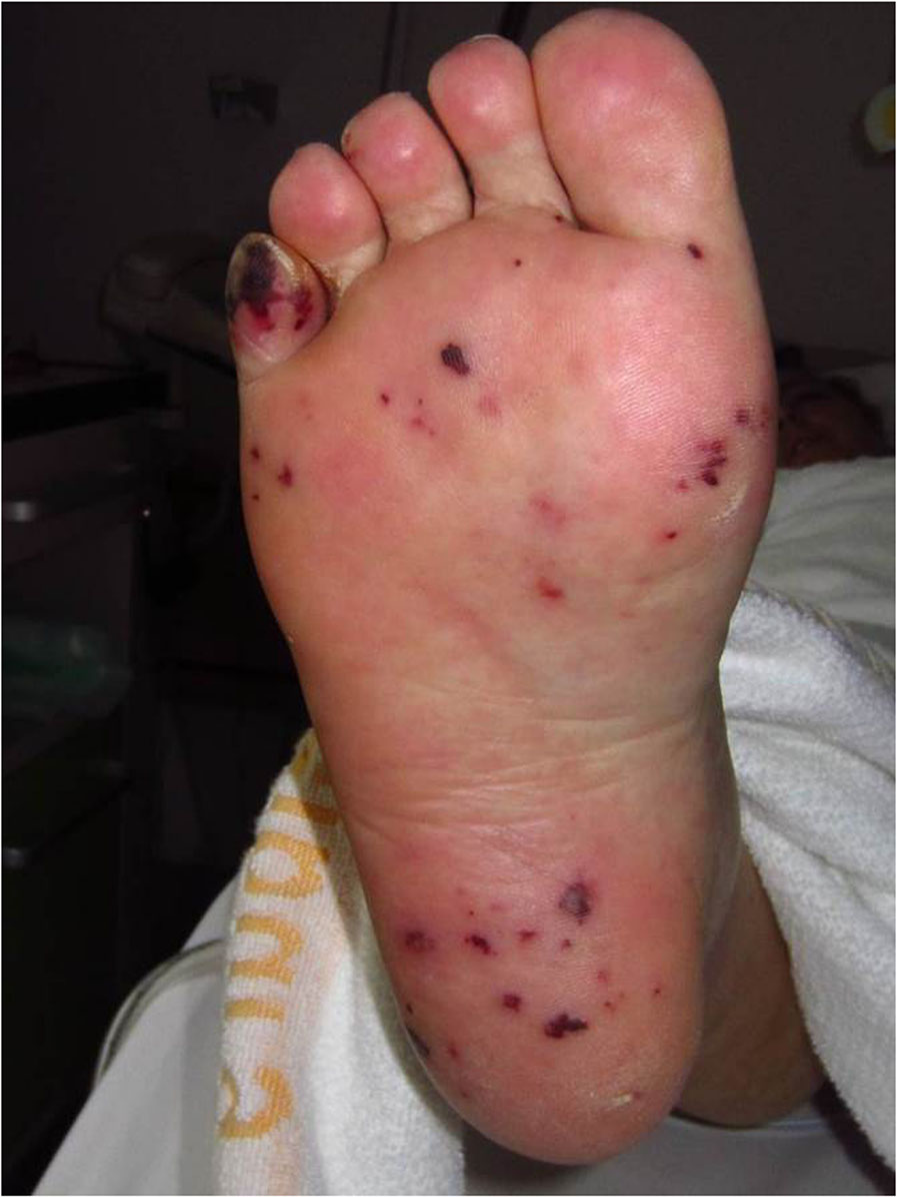
Multiple dark maculae on the sole of the right foot resembling septic emboli/Janeway lesions

**Fig. 3:**
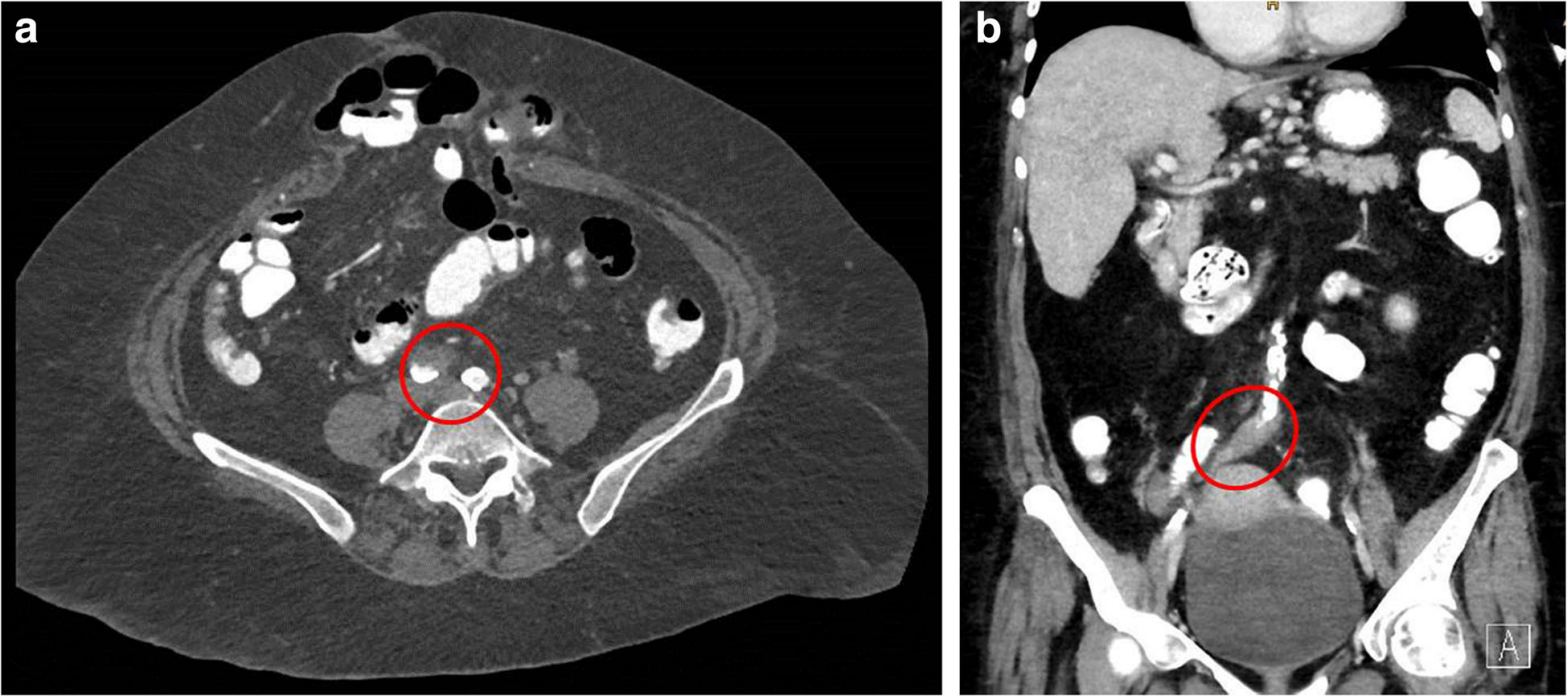
**a** and **b** CT scan showing an aortic wall thickening at the aortic bifurcation with a soft tissue cuff above the right common iliac artery consistent with endarteritis

**Fig. 4:**
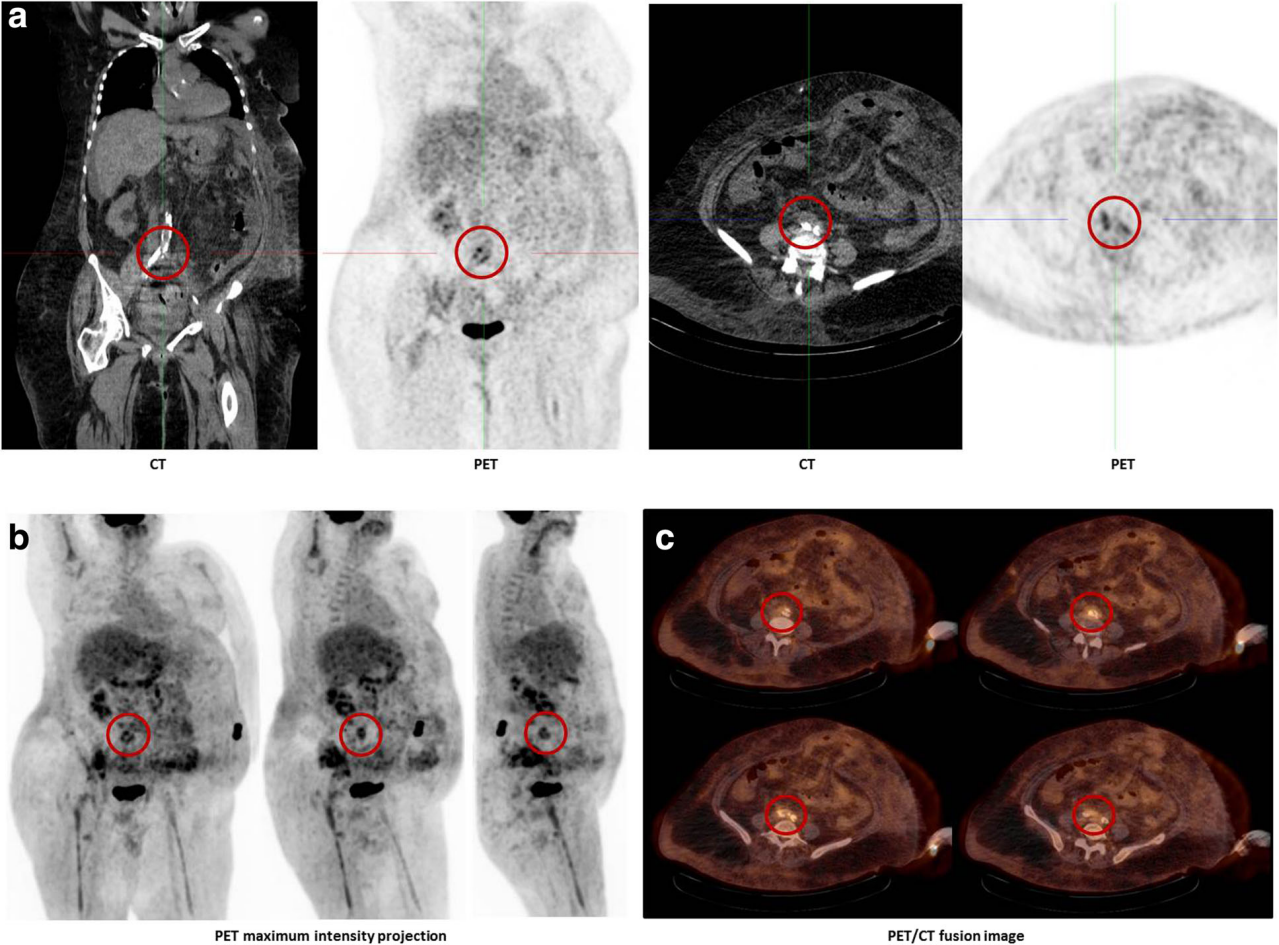
**a**-**c** FDG-PET/CT scan showing increased metabolism at the soft tissue cuff above the right common iliac artery

A 64-year old Caucasian woman presented in March 2014 to the emergency department with severe pain and swelling of her right foot. She also noticed small, dark lesions on the sole of her right foot. She denied fever but reported chills for several days (timeline see Fig. 1). No other symptoms were present. She had a history of plaque psoriasis and psoriatic arthritis since 2010. In the past she had received etanercept (stopped in 2010), adalimumab (stopped 2011), cyclosporine (stopped February 2013) and golimumab (stopped Oktober 2013) without significant clinical improvement. She was started on ustekinumab (45 mg subcutanously) in March 2014, received the second dose in April and the third dose three days before hospitalisation. One day before presentation, she had received a steroid injection in the right sacroiliac joint because of aggravation of pain in the lumbosacral region. Her medical history was remarkable for an alcohol induced liver cirrhosis (Child-Pugh stage B), arterial hypertension and breast cancer (stage pT1c, pN1a, M0; resection and radiation in 2006, since then in remission). On admission, the patient was afebrile (36.8 °C) and the vital signs were within normal ranges (blood pressure 140/80 mmHg, pulse 87/min). The clinical examination revealed swelling of her right ankle and multiple non-elevated dark maculae on the sole of her right foot (Fig. 2a and b) resembling Janeway lesions. All joints were tender on palpation with particular pain in the right sacroiliac joint. Multiple psoriatic lesions were present. Laboratory analysis revealed a leukocytosis of 23 × 10 [9] cells/L, a reduced platelet count of 66 × 10 [9] cells/L, a C-reactive protein level of 154 mg/L [<5 mg/L], a procalcitonin of 4.67 ng/mL [<0.5 ng/mL], a blood urea nitrogen of 93 mg/dl, a serum creatinine of 1.16 mg/dl and an INR 1.35. A CT scan on the day of admission showed no signs of arthritis, joint effusion or empyema in the right ankle joint. However, edema and thickening of the aortic/right common iliac artery wall at the aortic bifurcation, consistent with an inflammatory vascular lesion, was noted (Fig. 3a and b). Without an unambiguous focus (and prior to the CT scan) the patient had been started on an empiric antibiotic therapy with intravenous piperacillin/tazobactam (4.5 g every 8 h) and oral clarithromycin (500 mg/d). The next day, the patient’s condition improved. Blood cultures yielded growth of *S. aureus* (susceptible to methicillin, fluoroquinolones, clindamycin and rifampicin). Antibiotic treatment was modified to intravenous flucloxacillin (4 g every 8 h). Transesophageal echocardiography revealed no evidence for infective endocarditis. Rheumatoid factor was negative and the urine analysis did not show signs of proteinuria or hematuria. A follow-up CT scan 12 days later confirmed arterial wall thickening at the aortic bifurcation with a soft tissue cuff above the right common iliac artery with no evidence for progression. An additional FDG-PET/CT scan demonstrated increased metabolism in this vessel segment (Fig. 4a-c). Apart from moderate inflammatory activity in the right sacroiliac joint no other hypermetabolic foci were detected.

The patient was treated for four weeks with intravenous flucloxacillin and was discharged with an oral regime consisting of levofloxacin (500 mg/d) and rifampicin (450 mg twice daily) for another four weeks. One month after completion of the antibiotic therapy the patient was well with no signs of active infection or recurrence. A report was filed with the Federal Institute for Drugs and Medical Devices (BfArm).

## Conclusions

To our knowledge, this is the first published report of a severe *S. aureus* infection in a patient receiving ustekinumab. Ustekinumab is a rather new biological immunosuppressive agent that is used in moderate to severe plaque psoriasis and otherwise unresponsive active psoriatic arthritis. Its efficacy and safety has been studied in a multicenter, prospective, disease-based registry (Psoriasis Longitudinal Assessment and Registry [PSOLAR]) [12] as well as in multiple large multicenter trials [10, 13–16]. Ustekinumab is considered to be well tolerated, most adverse events are mild and do not require dosage adjustment or cessation of therapy [17]. Clinical trial data available so far indicate no increased incidence of severe infections compared to placebo or other biologicals and no specific patterns of infection could be observed [9, 11–13, 17, 18]. The PSOLAR registry included 4134 patients receiving ustekinumab. The cumulative incidence for serious infections was 0.95/100 patient years compared to 1.26 for non-biologicals and 2,78 for infliximab.

IL-12 plays an important role for the optimal production of IFN-γ by T cells and NK cells [19]. Patients with an inherited defect in the IFN-γ/IL-12 pathway show a decreased IFN-γ production due to a disturbed interaction between phagocytes and T lymphocytes and an increased susceptibility to infections with microorganisms of the genera *Mycobacterium* and *Salmonella* [20–22]. Severe *S. aureus* infections have not been documented so far in such patients or patients receiving ustekinumab. Of note, IL-23 stimulates Th-17 cell development. Recent evidence suggests that Th-17 cells contribute to cutaneous defense against *S. aureus* [23]. As ustekinumab indirectly inhibits Th-17 pathways this may be regarded as a theoretical and of course purely speculative explanatory model for a propensity/higher susceptibility for *S. aureus* infections. In general, patients receiving biologicals and other immunosuppressive agents for the treatment of rheumatologic disorders may be at increased risk for severe infections due to different reasons. In addition to the immunsupressive treatment, these patients are frequently exposed to injections for application of drugs but also for diagnostic or therapeutic intraarticular punctures. Moreover, psoriatic skin lesions have been shown to be more frequently colonized by *S. aureus* than healthy skin [24]. In our case of a healthcare-associated *S. aureus* bacteremia the portal of entry remains unclear. Potential entry sites include psoriatic skin lesions or intravascular catheters (which were present during previous hospitalisations). The inflammatory erosive changes of joints seen in psoriatic (or rheumatoid) arthritis may represent an independent risk factor for osteoarticular infections, as increased adherence to inflammatory altered joint surfaces likely accentuates seeding of pathogens such as *S. aureus* [25–27].

However, we found no clear evidence for *S. aureus* infection of the sacroiliac joint secondary to injection therapy. Moreover, extension from a contiguous focus was considered highly unlikely as surrounding tissue was without pathological findings in several radiological examinations.

Endarteriitis as seen in our case represents a rare diagnosis in the absence of endovascular prosthesis or preexisting aneurysm. However, hematogenous seeding in the endothelium of an otherwise intact arterial intima during bacteremia may occur and *S. aureus* is the predominant pathogen in this context. Our patient had a number of risk factors that might have contributed to the observed severe infection like psoriatic lesions, injection therapy and pior hospitalizations. Besides ustekinumab, our patient had received a number of different immunosuppressive drugs in the past, however, the latest 5 month prior to the reported infection. Of course, in these complex clinial setting, it is not possible to unambiguously assign ustekinumab as the underlying cause for this infection and this is not the intention of our report. However, we believe that in terms of pharmacovigilance, reporting of such cases is important.

## Abbreviations

BfArm: Bundesanstalt für Arzneimittel und Medizinprodukte (Federal Institute for Drugs and Medical Devices)
BSI: Blood stream infection
EMA: European Medicines Agency
FDA: Federal Drug Agency
PSOLAR: Psoriasis Longitudinal Assessment and Registry
SAB: *Staphylococcus aureus* bacteremia
TNF-α: Tumor necrosis factor alpha
